# The perspective of gender on the Ebola virus using a risk management and population health framework: a scoping review

**DOI:** 10.1186/s40249-017-0346-7

**Published:** 2017-10-11

**Authors:** Miriam N. Nkangu, Oluwasayo A. Olatunde, Sanni Yaya

**Affiliations:** 1 School of Epidemiology, Public Health and Preventive Medicine, University of Ottawa, 451 Smyth Road, RM 2016, Ottawa, K1H 8M5 Canada; 2 Department of Family Medicine, Dalhousie University, Halifax, 208–44 Mapleton Road, Moncton, NB E1C 7W8 Canada; 3 School of International Development and Global Studies, University of Ottawa, 120 University, Social Sciences Building, RM 8005, Ottawa, K1N 6N5 Canada

**Keywords:** Ebola, Gender and Ebola virus disease, Global health, Women and Ebola virus disease, Women and care giving roles, Ebola and hunting of bush meat, men and hunting of bush meat

## Abstract

**Background:**

In the three decades since the first reported case of Ebola virus, most known index cases have been consistently traced to the hunting of “bush meat”, and women have consistently recorded relatively high fatality rates in most catastrophic outbreaks. This paper discusses Ebola-related risk factors, which constantly interact with cultural values, and provides an insight into the link between gender and the risk of contracting infectious diseases, using Ebola virus as an example within Africa.

**Method:**

A comprehensive search of the literature was conducted using the PubMed, Ovid Medline and Global Health CABI databases as well as CAB Abstracts, including gray literature. We used a descriptive and sex- and gender-based analysis to revisit previous studies on Ebola outbreaks since 1976 to 2014, and disaggregated the cases and fatality rates according to gender and the sources of known index cases based on available data.

**Results:**

In total, approximately 1530 people died in all previous Ebola outbreaks from 1976 to 2012 compared with over 11,310 deaths from the 2014 outbreak. Women’s increased exposure can be attributed to time spent at home and their responsibility for caring for the sick, while men’s increased vulnerability to the virus can be attributed to their responsibility for caring for livestock and to time spent away from home, as most known sources of the index cases have been infected in the process of hunting. We present a conceptual model of a circle of interacting risk factors for Ebola in the African context.

**Conclusion:**

There is currently no evidence related to biological differences in female or male sex that increases Ebola virus transmission and vulnerability; rather, there are differences in the level of exposure between men and women. Gender is therefore an important risk factor to consider in the design of health programs. Building the capacity for effective risk communication is a worthwhile investment in public and global health for future emergency responses.

**Electronic supplementary material:**

The online version of this article (doi:10.1186/s40249-017-0346-7) contains supplementary material, which is available to authorized users.

## Multilingual abstract

Please see Additional file 1 for translations of the abstract into five official working languages of the United Nations.

## Background

Gender is a determinant of health that has been given relatively little attention in medicine and in the design of national and global health programs [1]. When gender is considered, it is most often from the perspective of women rather than both men and women. It is therefore important to distinguish between gender and sex, as both terms have been used inappropriately in the literature [1, 2]. Sex refers to the biological characteristics of men and women, while gender denotes the socially constructed characteristics of men and women, which are attributed to a specific culture and context and change over time [1–4]. In the context of Ebola, sex-disaggregated data serve to analyze gender as a determinant of health but could also help stimulate ideas on incorporating gender into health planning and intervention programs for the utilization of health services. This is particularly important as Ebola-related risk factors are associated with specific gender roles and therefore interact with cultural values within the African context.

In defining gender-related differences, the World Health Organization (WHO) describes how gender roles “influence where men and women spend their time, and the infectious agents they come into contact with, as well as the nature of exposure, its frequency and its intensity,” and “differences influence the course and outcome of disease for those who have been infected” [4]. The WHO further highlights common differences in gender roles that influence exposure patterns, including the following: (i) time spent at home and away from home; (ii) responsibility for caring for the sick; (iii) responsibility for caring for livestock; (iv) access to healthcare; and (v) scientific knowledge about treatment [4]. These gender differences and their association with Ebola-related risk factors are discussed at the end of this paper to connect gender and Ebola disease in Africa.

Fruit bats are thought to be the primary host of the Ebola virus [5], and most sources of known index cases of Ebola since the first outbreak in 1976 have been consistently traced to exposure to “bush meat” [5]. Bush meat is encountered across most parts of Africa and refers to wild animals in the forest or non-domestic animals. Examples of such wild animals include the following: gorillas, chimpanzees, forest antelopes (duikers), porcupines, and crocodiles. The first known case of Ebola outbreak in Yambuku, Democratic Republic of Congo (DRC), was a 44-year-old male teacher known to have purchased fresh and smoked antelope and monkey (bush meat) approximately 50 km north of Yambuku and had also eaten stewed antelope [6]. Thus, this paper uses the term “bush meat” to reflect the local reality and culture. The hunting of bush meat is an occupation and an activity that is culturally associated with men within the African context. Hence, bush meat is a source of protein and is also considered a source of income and livelihood. Moreover, the consumption of bush meat is not linked to differences in socio-economic status in Africa.

**Fig. 1:**
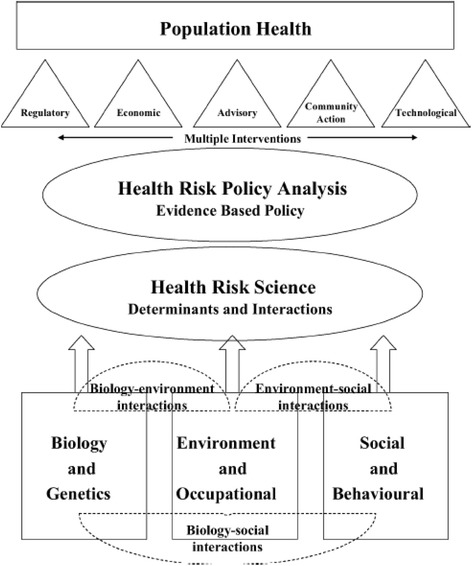
An integrated framework for risk management and population health,Krewski et al. (2007). Reproduce with Permission from Taylor and Francis Group. Ref.P062817–01.. Source: Daniel Krewski, Victoria Hogan, Michelle C. Turner, Patricia L. Zeman, Ian McDowell, Nancy Edwards and Joseph Losos. “An Integrated Framework for Risk Management and Population Health,” Human and Ecological Risk Assessment: An International Journal, 2007, 13, (6)

**Fig. 2:**
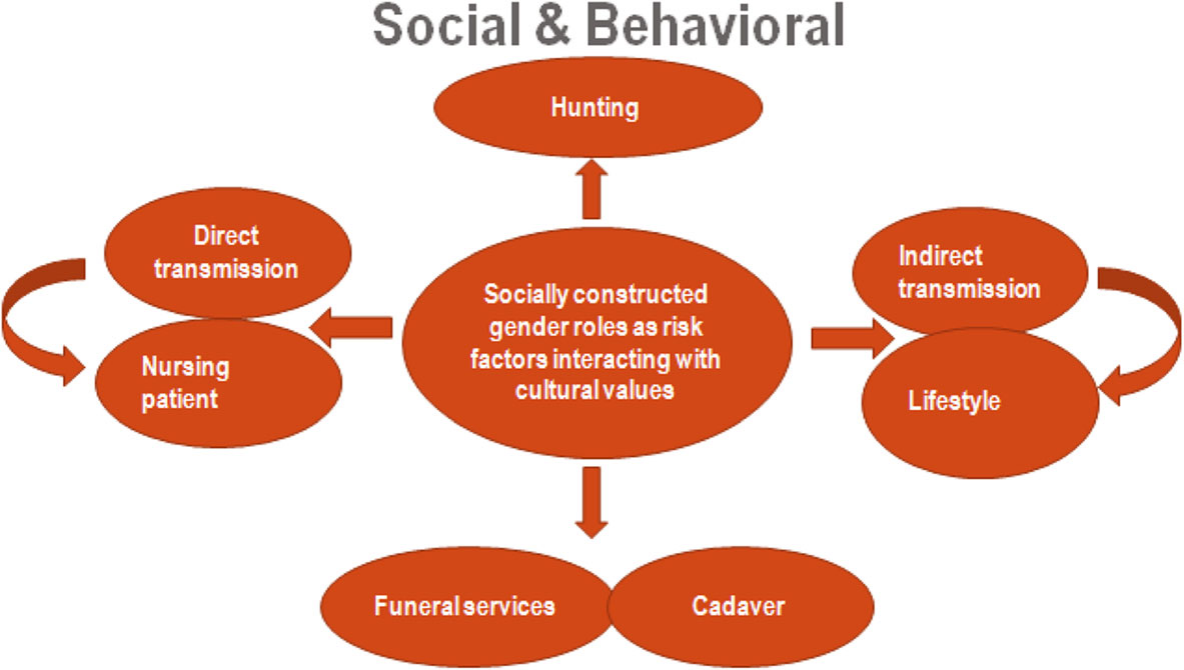
Gender roles as risk factors and cultural values- a circle of interacting risk factors [42]

Women are typically considered the primary caregivers during illness. In their attempt to fulfill their gender roles, women are more inclined to nurse children and care for their sick husbands, sisters, and brothers as well as their entire support network. Fulfilling these duties becomes a responsibility for women. In contrast, it is uncommon for men to take care of their wives or children when they are ill, as this role is often assumed by other family members or children if they are of “reasonable age”. Given that women are at higher risk of exposure due to their gender roles, their support network is also at risk. This paper uses a sex- and gender-based analysis approach [2] as well as a risk management and population health framework developed by Krewski et al. (2007) (see Fig. 1) to categorize gender-associated risk factors. In addition, we present a conceptual analysis of a circle of interacting risk factors which illustrate how gender-related risk factors interact with cultural values (see Fig. 2). Given that not all risk can be managed at the domestic level and because risk assessment informs subsequent risk management, this paper will direct and inform health authorities and global health policy-makers regarding how to consider gender when planning for and managing future Ebola outbreaks. Thus, the objective of this study was to provide insight into the link between gender and the risk of contracting infectious diseases using Ebola virus as an example within Africa.

## Methods

We used a descriptive and sex- and gender-based analysis (SGBA) to revisit previous studies on Ebola outbreaks since 1976. A sensitive and comprehensive search of the literature was conducted in the PubMed, Ovid Medline, and Global health CAB databases, as well as the gray literature. Ovid Medline and Global Health CAB were searched using the following MeSH terms: “Ebola hemorrhagic fever,” “Ebola,” “sex,” “male and female,” “gender,” and “viral hemorrhagic fever.” After this initial pilot search, which yielded 679 publications, we excluded the term “viral hemorrhagic fever,” which was the index term used from 1978 to 1995 in MEDLINE, because of noise. We retained 469 publications and 39 publications were included in the final review (see supplemental for details). All titles and abstracts were screened to identify original articles that reported the outcomes of actual human Ebola outbreaks, including confirmed cases, case fatality rates, and/or sex or gender. The search was extended by inspecting the references of selected articles. We reviewed Ebola outbreaks from 1976 to 2014 and disaggregated the cases and fatality rates according to sex; we also identified the sources of known index cases based on available data.

We employed the population health risk management framework described by Krewski et al. (2007) to characterize the risk. The framework illustrates how “population health enhances health through multiple interventions by modifying health determinants and the interactions among them, whereas risk management strives for risk avoidance by mitigating exposure to individual risk factors that can lead to adverse health outcomes” [7]. We used the risk assessment component of the framework to explore reported risk factors for Ebola and to inform risk management and planning. As such, we emphasize social and behavioral considerations and present the concept of a “circle of interacting risk factors”. Finally, we discuss the relevance of the advisory and community components of the risk management aspects of the framework and highlight the importance of effective risk communication as a tool in this context.

## Results

In total, approximately 1530 people died in all Ebola outbreaks from 1976 to 2012, compared with over 11,310 deaths in the 2014 outbreak (data as of April 2016) [8]. Since the first outbreak in 1976, all the sources of known index cases of Ebola (see Table 1) have been traced to the hunting of bush meat or exposure to dead animals in the rainforest [5]. A relatively high fatality rate has been consistently recorded among women in most of the catastrophic outbreaks [4, 6, 9]. In the 1976 outbreak in the DRC, the mortality rate was 56% among women and 44% in men [6]. Similarly, of the 315 cases reported in a 1995 nosocomial outbreak, 53% were in women, and 47% were in men [9]. In the 2014 outbreak, more cases were recorded among women than men [8, 10]. In Nigeria, women accounted for 55% of the cases, and men accounted for the remaining 45% [11].

**Table 1:** Sex distribution of Ebola virus cases and the exposure type of known index cases from 1976 to 2014

Source	Year	Country	Ebola Species	Sources of known index cases	Cases (*n*)		Total Cases	Fatality Ratio %	
					Males	Females		Males	Females
Bulletin of WHO, 1978	1976	Sudan	Sudan virus	Unknown	x	x	284	56	48^a^
Bulletin of WHO, 1978	1976	DRC	Zaire virus	Unknown	141	177	318	44	56
Heymann et al., 1980, & Leroy et al., 2009	1977	Tandala, DRC	Zaire virus	Unknown	0	0	1	0	100
Baron et al., 1983 [27]	1979	Sudan	Sudan virus	Unknown	13	21	34 (22 deaths)	x	x
Leroy et al., 2009, CDC, 2016	1994	Ivory Coast	Tai Forest virus	Chimpanzee	1	0	1 (no deaths)	0	0
Georges et al., 1999 [28]	1994	Gabon	Zaire virus	Chimpanzee, Gorilla	x	x	52 (31 deaths)	x	x
Muyembe-Tamfum et al., 1999 & Roels et al., 1999 [29]	1995	DRC	Zaire virus	Unknown	149	166	315	50.4	49.6
Georges et al., 1999	1996	Gabon (Spring)	Zaire virus	Unknown	17	14	31 (21 deaths)	x	x
Georges et al., 1999	1996	Gabon (Fall)	Zaire virus	Chimpanzee	x	x	60 (45 deaths)	x	x
CDC 2014 [30], Francesconi et al., 2003 [31], & Lamunu et al., 2003	2000	Uganda	Sudan virus	Unknown	156	269	425 (224 deaths)	x	x
Leroy et al., 2009 & Nkoghe et al., 2011	2001–2002	Republic of Congo	Zaire virus	Chimpanzee, Gorilla, Monkey	x	x	59 (44 deaths)	x	x
Nkoghe et al., 2005 & Leroy et al., 2009	2001–2002	Gabon	Zaire virus	Chimpanzee, Gorilla	34	31	65 (53 deaths)	x	x
Boumandouki et al., 2005 [32], Nkoghe et al., 2011 [33], & Leroy et al., 2009 & 2005 [34]	2003	Republic of Congo	Zaire virus	Gorilla, Monkey	x	19	35 (29 deaths)	x	x
Formenty et al., 2003 [35], Leroy et al., 2009 & Nkoghe et al., 2011	2003 (Jan-June)	Republic of Congo	Zaire virus	Gorilla, Monkey	53%(patient)	47	143 (128 deaths)	x	x
Nkoghe et al., 2011	2005 (April–May)	Republic of Congo	Zaire virus	Handling animals	10	2	12 (10 deaths)	x	x
Grard et al., 2011 [36] & CDC, 2016	2007–2008	DRC	Zaire virus	Unknown	x	x	264 (187 deaths)	x	x
		Uganda	Bundibugyo virus	Unknown	x	x	149 (37 deaths)	x	x
Leroy et al., 2009	2007	Uganda	Bundibugyo virus	Unknown	x	x	116 (30 deaths)	x	x
CDC, 2016 [37]	2012	DRC	Bundibugyo virus	Unknown	x	x	36 (13 deaths)	x	x
WHO, 2014, Nanclares et al., 2016 [38], & Maganga et al., 2014 [39]	2014	DRC	Zaire virus	Gorilla, Monkey	x	x	66 (49 deaths)	x	x
WHO, 2016 (data as of May 2016)	2014	Guinea	Zaire virus	Unknown	1599	1747	3346	x	x
		Sierra Leone		Imported	4823	5118	9941	x	x
		Liberia		Imported	1911	1838	3749	x	x

^a^Indicates data as reported in the article

x Indicates that data were not available in disaggregated form

Reported data included both probable and confirmed cases

### Gender, household, and hospital transmission

A systematic review conducted by Brainard et al. (2016) found the risk of transmission to be higher for those caring for the sick at home (unadjusted PPR 13.33, 95% *CI*: 3.2–55.6). In most Ebola outbreaks, the transmission rate has been higher in households than in hospitals, [9, 12–14]. For example, in the 1976 outbreak in Sudan (Nzara and Maridi), 58% of infections were traced to household contacts, and 35% were traced to hospital settings [12]. A study conducted by the WHO in 2007 reported a predominance of men in the early stages of the 2001–2002 outbreaks in Gabon and Congo, whereas women outnumbered men during the later stages of the outbreaks. In contrast, in the 2000–2001 outbreak in Uganda, the number of female cases exceeded the number of male cases throughout the outbreak [4]. These trends are not well understood [4]. In a recent study conducted by the WHO Ebola response team to assess sex differences among 20,035 cases reported in the three most affected countries (Guinea, Liberia, and Sierra Leone) during the 2014 outbreak, females and males had a similar average risk of contracting the virus [10]. Although the frequency of exposure were higher among women than men (34.3%, 95% *CI*: 33.4–35.2 vs. 30.7%, 95% *CI*: 29.8–31.7; *P* < 0.001), and women reported more exposure during funerals than men, female patients had a higher survival than male patients, and the odds of death were lower for females than for males after adjusting for age (*OR*: 0.83, 95% *CI*: 0.76–0.91) (see ref. [10], supplemental appendix) [10]. Francesconi et al. (2003) also found that neither age (> 30 years vs. ≤ 30 years: prevalence proportion ratio (PPR) = 1.38, 95% *CI*: 0.64–2.97) nor sex (female vs. male: PPR = 1.54, 95% *CI*: 0.66–3.60) was significantly associated with the risk of contracting Ebola [15]. Similarly, the WHO Ebola response team found that exposure did not vary by age in the 2014 outbreak [10]. In the 2014 outbreak, the average interval from symptom onset to hospitalization was 0.5 days shorter in female patients than in male patients in all three of the most affected countries [10]. The proportion of male patients was not significantly different from the proportion of males in the general population of the respective countries, except for one specific district, Gueckedou (Guinea), which had a very low proportion of male patients [10], this variation was not explained.

### Gender roles as risk factors -a circle of interacting risk factors

The circle of interacting risk factors provides insight into the interaction between Ebola risk factors and socially constructed gender roles where the direct transmission of Ebola virus occurs through contact with infected patients, dead bodies, or bodily fluids [14, 15]. Indirect transmission may occur when sharing meals, washing clothes, sleeping in the same bed, sharing clothing, shaking hands, or hugging, as well as during ritual hand washing and communal meals at funerals [14–16]. Dead bodies carry a high viral load [14, 17], and cultural practices associated with funerals put both men and women at high risk. Men of high societal status and those who engage in some religious practices may be required to touch dead bodies and dress them, and women may be required to bathe, dress, shave, and touch dead bodies as part of the traditional rites performed during such ceremonies [13]. Both men and women have specific cultural roles during funeral services. For example, in the outbreak in Gabon in 2001–2002, women took care of the dead bodies of women, and men took care of the dead bodies of men, according to their tradition [13]. All these risk factors and related exposures interact with cultural values as shown in Fig. 2.

## Discussion

There is currently no evidence related to biological differences in female or male sex that increases Ebola virus transmission and vulnerability; rather, there are differences in the level of exposure between men and women [10, 11, 18].. Data from the 2014 outbreak (December 2013 – August 2015) suggested that female patients with confirmed Ebola were less likely to die than male patients [10]. This finding is significant when considering the large number of cases in the study, which was powered to detect small differences in outcomes. However, given that most of the previous outbreaks did not often report cases and fatality by sex, it is important to conduct further research using a sex- and gender-based analysis approach [2].

### Gender differences that influence exposure patterns

Ebola outbreaks require an emergency response, and pre-existing knowledge and understanding of exposure patterns and their interplay with gender-associated risk factors provide fundamental assistance with planning such a response. Below, we discuss these differences and gender-related risk factors in more detail, using available evidence to inform health policy.

### Responsibility of caring for livestock and time spent away from home

Most known index cases in epidemiological reports have been traced to hunting of or exposure to bush meat [5]. However, there has been little effort to help those who are responsible (men) for this activity in performing their role (hunting of bush meat). Although women are sometimes involved in cross-border trading, which may increase their level of exposure and could be considered as time spent away from home, data on source of known index cases point to the importance of hunting as a catalyst of outbreak. There is a possible connection between patterns of time spent away from home taking care of livestock and the finding that most source of index cases have been traced to hunting. The primary healthcare (PHC) movement advocated for “community participation” [1] but ignored gender roles [1, 19], and the move away from comprehensive PHC to selective PHC further hindered this goal. In line with the health in all policies approach, global response strategies should, within a specific context, identify the various high-risk groups, establish the needs of the local community, and incorporate these factors into health planning programs.

### Time spent at home and responsibility for caring for the sick

The risk of transmission is found to be higher for those caring for the sick at home [14]. In the DRC outbreak in 1976, the high transmission rate reported in hospitals was due to the use of syringes [6]. A direct connection can be drawn between time spent at home and caring for the sick and the level of exposure and susceptibility. Women are considered caregivers and take on the role of “nurses” in their homes. They perform tasks that, to some extent, are similar to those performed by nurses in the hospital. However, nurses are trained and accredited, unlike informal caregivers, little attention is paid to informal caregivers when designing health programs within a specific context. This is further discussed in Table 2 using a case study in Liberia as an example.

**Table 2:** The Liberian case

On September 25, 2014, CNN posted on their website a story about a Liberian lady who took care of her entire family—mother, sister, father, and cousin—all of whom were infected. She fed and cleaned them and administered their medication all by herself. She invented her own personal protective equipment (PPE) using local materials known as “trash bags” [40] but did not get infected. Out of 4 patients she nursed, only one died. One may argue that, unlike most informal caregivers, she had some formal nursing training and knowledge, although she had not yet graduated.
The focus of this example is to expand on the following: (i) to illustrate a typical example of gender-specific roles in the African context; (ii) to show how women’s role and their task as caregivers are similar, to some extent, to those of nurses in a hospital setting; and (iii) to emphasize the importance of considering these gender roles when planning and designing health programs within a specific context. http://www.cnn.com/2014/09/25/health/ebola-fatu-family/) [41].

### Scientific knowledge regarding treatment and access to healthcare

In the absence of a licensed treatment for Ebola at the time that this paper was prepared, we use the advisory and community components of the framework by Krewski et al. *Human and Ecological Risk Assessment: An International Journal* (2007) to discuss the scientific knowledge and access to care in the context of Ebola, focusing on risk perception and effective risk communication.

Ebola virus has always been perceived in the community as either a “mysterious illness” or “witchcraft” [6, 20]. Although risk perception by the general public has always been at odds with expert opinion [21], risk perception varies by context, gender, and level of education, all of which may also influence care-seeking behavior. There is little to no data on the level of education by gender among Ebola-infected patients or survivors. However, evidence from the literature indicates that risk perception varies by gender and level of education [21], both of which impact access to health services [22]. For example, in a systematic review of gender-related barriers to accessing treatment for tuberculosis (TB), an infectious disease characterized by stigmatization, Krishnan et al. (2014) found that low education in women correlated with greater fear of contracting TB, that men had more knowledge about TB transmission than women, and that socio-cultural norms associated with the status of men and women directly affected the types of barriers encountered while accessing treatment [22]. Most women in Africa, especially those who are less educated, do not feel they have the right to refuse sex once they are married, and there is little awareness that men can transmit the virus through semen for up to seven weeks after recovery [17, 23].

In the 1995 outbreak, some survivors accepted that Ebola is a preventable disease, and some considered it a divine punishment from God [24]. Public health messaging during the 2014 outbreak emphasized that “Ebola is real”, with repeated messages that “it is deadly and has no cure, no treatment, and no vaccine” [20]. While this advisory message sought to inform an “ignorant” community that the disease has no cure, it may have had a perverse effect: people in the community were advised to seek immediate care in the hospital in the event of any symptoms, but they witnessed patients going into the hospital for treatment and dying. This calls to mind the work of Jardine and Hrudley (1997), who found that mixed messages in risk communication caused differences in understanding and interpretation between the risk managers and the affected population [25]. Such messages may lead to differences in processing and understanding of the risk message, especially when technical or scientific terms are used, such as “no cure, no treatment, and no vaccine”, in addressing a lay population with multiple spoken languages and dialects. Moreover, gender differences in the use of services may arise depending on several factors: To whom and in what language is the message actually delivered? How is the message delivered? Who actually makes the decision to seek care? Who makes the decision to take a patient away from the hospital? Who actually cares for the patient? A WHO report clearly stated that “when technical interventions cross purposes with entrenched cultural practices, culture always wins” and that “by implication, control efforts should work within the culture and not otherwise” [20].

### Limitations

There have been discrepancies in data collection and variations in data reporting across the various Ebola outbreaks that have occurred since 1976. For instance, most of the data collected did not systematically take sex and gender into consideration; thus, the data could not be disaggregated. This explains why some of this information is not provided in Table 1. This is in line with Harman’s explanation that “discrepancy over the data reported during Ebola outbreaks is problematic for the visibility of women and gender” [26]. We found that most studies did not collect or report information about the level of education of infected patients or survivors by gender. These data may have been collected by health authorities but were infrequently reported in a disaggregated form in most studies. We do not report case fatality for the 2014 outbreak in Table 1 because the data were not yet available at the time of the preparation of this manuscript. We focused on gender roles and thus did not consider pregnancy related risk factors, which we consider to be more biological and pregnancy does not change any aspect of gender roles. Furthermore, we did not include age, because there was no significant difference by age in disease exposure or outcomes [10, 15]. Moreover, gender roles in the African context do not depend on age per se but rather on whether a person is of “reasonable age” to undertake a given role. Finally, although health workers are generally at higher risk of contracting the virus, we did not discuss the impact of gender in this population and occupation. Nevertheless, it is important to note that women often comprise the majority of nurses in the hospital. Women are often given lower status and recognition, and most families prefer to train and educate male children as doctors and female children as nurses (if given the opportunity) based on perceived gender roles. As a consequence, women serve more often than men as frontline caregivers in the hospital.

## Conclusion

It is important to disaggregate data by sex, which can help inform gender-related research, health planning and policies. The need to build the capacity for effective risk communication as a worthwhile investment for both local and global public health authorities, and to understand and respect subtle cultural and socio-economic undertones relating to gender should not be undermined. The hope is that global and national health policies will better incorporate gender-based lessons drawn from the following: (i) the consistent tracing of the source of known index cases of Ebola to the hunting of bush meat, (ii) the high rate of transmission in the household and during burials, and (iii) gender-related differences and interaction in exposure patterns and risk factors.

## Additional file

Additional file 1: Multilingual in the five official working languages of the United Nations. (PDF 580 kb)

## Abbreviations

DRC: Democratic Republic of Congo
PPR: Prevalence proportion ratio
TB: Tuberculosis
WHO: World Health Organization
ZBOV: Zaire Ebola Virus
