# Supplementary material for: The perspective of gender on the Ebola virus using a risk management and population health framework: a scoping review
Source: Infect Dis Poverty. 2017 Oct 11;6:135. doi: 10.1186/s40249-017-0346-7 (PMC5635524; doi:10.1186/s40249-017-0346-7)
Supplement: Additional file 1: — Multilingual in the five official working languages of the United Nations. (PDF 580 kb) [file 40249_2017_346_MOESM1_ESM.pdf]

Translation of the abstract into the five official working languages of the United Nations

منظور نوع الجنس على فيروس إيبولا باستخدام إطار إدارة المخاطر والصحة السكانية: نظرة شاملة.

ميريام ن. نكانغو, أولواسايو أ. أولاتوند, ساني يايا

ملخص

خلفية: في العقود الثلاثة التي انقضت منذ أول حالة تم الإبلاغ عنها عن فيروس إيبولا، كانت معظم الحالات المسجلة المعروفة تصيب باستمرار من يصطادون "لحوم الأدغال"، وسجلت النساء دائما معدلات مرتفعة نسبيا من الوفيات في معظم حالات تفشي المرض الكارثية. وتناقش هذه الورقة عوامل الخطر المرتبطة بالإيبولا، التي تتفاعل باستمرار مع القيم الثقافية، وتوفر نظرة فاحصة للصلة بين نوع الجنس وخطر الإصابة بالأمراض المعدية، باستخدام فيروس إيبولا كمثال في أفريقيا.

الطريقة: أجري بحث شامل في الكتابات عن هذا الموضوع باستخدام قواعد بيانات ببمد (PubMed) و أوفيد مدلين (Ovid Medline) و غلوبال هيلث كابي (Global Health CA) بالإضافة إلى ملخصات كاب (CAB)، بما في ذلك الكتابات والابحاث غير الرسمية. استخدمنا تحليلا وصفيا وقائما على الجنس لإعادة النظر في الدراسات السابقة حول تفشي الإيبولا منذ 1976 إلى 2014، وصنفنا الحالات ومعدلات الوفيات حسب نوع الجنس ومصادر الحالات المسجلة المعروفة استنادا إلى البيانات المتاحة.

النتائج: في المجموع، توفي ما يقرب من 1530 شخصا في جميع حالات تفشي الإيبولا السابقة من 1976 إلى 2012 مقارنة مع أكثر من 11310 حالة وفاة عام 2014 بسبب الإيبولا. ويمكن أن يعزى زيادة تعرض النساء للمرض إلى الوقت الذي يقضيهنه في المنزل ومسؤوليتهن عن رعاية المرضى، في حين يمكن أن يعزى زيادة تعرض الرجال للفيروس إلى مسؤوليتهم عن رعاية الماشية وإلى الوقت الذي يقضونه بعيدا عن المنزل، حيث أن معظم مصادر الحالات المسجلة تعرضت للاصابة في عملية الصيد. نقدم نموذجا مفاهيميا لدائرة من عوامل الخطر المتفاعلة للإيبولا في السياق الأفريقي.

خاتمة: لا يوجد حاليا أي دليل على وجود اختلافات بيولوجية في جنس الإناث أو الذكور مما يزيد من انتقال فيروس إيبولا والتعرض له؛ بدلا من ذلك، هناك اختلافات في مستوى التعرض بين الرجال والنساء. ومن ثم، فإن نوع الجنس هو عامل خطر وهام ينبغي النظر فيه عند تصميم البرامج الصحية. ويعد بناء القدرة على التواصل الفعال للمخاطر استثمارا مجديا في الصحة العامة والعالمية من أجل الاستجابات الطارئة في المستقبل.

Translated from English version into Arabic by Mohamed Sahal, through

TRANSLATORS WITHOUT BORDERS

使用风险管理和人群健康框架评估性别因素在埃博拉病毒中的作用：勘域综述

Miriam N. Nkangu, Oluwasayo A. Olatunde, Sanni Yaya

摘要

引言：自首例埃博拉病毒病例报道已过去 30 年，多数已知病例可以一直追溯到“丛林肉（bush meat）”的狩猎活动。而在大多数灾难性疫情中，女性的致死率一直居高不下。本文以非洲的埃博拉病毒为例，探讨了与文化价值观念相关的病毒传播风险因素，并对性别和疾病易感性之间的联系进行分析。

方法：从 PubMed，Ovid Medline 和 Global Health CABI 数据库（包括灰色文献）、以及 CAB 文摘库中综合检索文献。基于性别的描述性分析回顾了 1976-2014 年间埃博拉暴发疫情的相关文献，根据已有资料基于性别和已知因素对病例和死亡率进行分类分析。

结果：1976-2012 年所有埃博拉暴发疫情共导致约 1 530 例患者死亡，而 2014 年暴发疫情的死亡病例超过 11 310 例。女性的暴露性增加可归因于室内活动和照顾病人的责任，而男性暴露性增加可归因于照顾家畜和户外活动，因为大多数病例是在狩猎过程中被感染。我们提出了一个埃博拉在非洲的背景下风险因素相互作用的概念模型。
结论：目前，没有证据证实性别在增加埃博拉病毒传播和易感性上存在生物学差异。然而，不同性别之间暴露程度存在差异，因此性别在涉及卫生项目时是重要的风险因素。对于公共卫生和全球健康而言，在未来应急响应中构建有效的风险交流能力是非常有价值的投资。

Translated from English version into Chinese by Peng Song, edited by Pin Yang

CHINESE CENTER FOR DISEASE CONTROL AND PREVENTION
NATIONAL INSTITUTE OF PARASITIC DISEASES

Le point de vue de genre sur le virus Ebola en utilisant un cadre de gestion des risques et de santé de la population: un examen de la portée

Miriam N. Nkangu, Oluwasayo A. Olatunde, Sanni Yaya

Résumé
Contexte: Au cours des trois décennies écoulées depuis le premier cas signalé du virus Ebola, les cas les plus répertoriés ont invariablement été attribués à la chasse à la «viande de brousse» et les femmes ont constamment enregistré des taux de mortalité relativement élevés dans la plupart des épidémies catastrophiques. Cet article traite des facteurs de risque liés à l'Ebola, qui interagissent constamment avec les valeurs culturelles, et donnent un aperçu du lien entre le genre et le risque de contracter des maladies infectieuses, en utilisant le virus Ebola comme exemple en Afrique.
méthode: Une recherche complète de la littérature a été menée en utilisant les bases de données PubMed, Ovid Medline et Global Health CABI ainsi que les résumés CAB, y compris la littérature non publiée. Nous avons utilisé une analyse descriptive et axée sur le sexe et le genre pour revoir les études antérieures sur les épidémies d'Ebola de 1976 à 2014 et a désagréger les cas et les taux de mortalité selon le sexe et les sources des cas recensés en fonction des données disponibles.
Résultats: Au total, environ 1 530 personnes sont mortes dans toutes les épidémies antérieures d'Ebola de 1976 à 2012, contre plus de 11 310 décès lors l'épidémie de 2014. L'exposition accrue des femmes peut être attribuée au temps passé à la maison et à leur responsabilité de prendre soin des malades, alors que la vulnérabilité accrue des hommes au virus peut être attribuée à leur responsabilité de prendre soin du bétail et du temps passé loin de chez eux, car la plupart des cas recensés ont été infectés dans le processus de chasse. Nous présentons un modèle conceptuel d'un cercle de facteurs de risque interactifs pour Ebola dans le contexte africain.
Conclusion: Il n'existe actuellement aucune preuve liée aux différences biologiques dans le sexe féminin ou masculin qui augmente la transmission et la vulnérabilité au virus Ebola; En fait, il existe des différences dans le niveau d'exposition entre les hommes et les femmes. Le genre est donc un facteur de risque important à prendre en compte dans la conception des programmes de santé. Le renforcement de la capacité de communication efficace des risques est un investissement utile dans la santé publique et mondiale pour les futures réponses d'urgence.

Translated from English version into French by Maud75, through

Оценка гендерного фактора в распространении вируса Эбола с помощью инструментов управления рисками и стандартов здоровья населения: обзор

Мириам Н. Нкагу, Олувасайо А. Олатунде, Санни Яя

Аннотация

Общая информация: За три десятилетия, прошедшие с момента первого случая заболевания вирусом Эбола, наиболее известные случаи первичного инфицирования последовательно отслеживались до охоты на дичь, при этом в большинствн катастрофических вспышек регистрировались относительно высокие показатели летальности среди женщин. Данная работа посвящена связанным с вирусом Эбола факторам риска, находящимся в постоянном взаимодействии с культурными ценностями, кроме того, она дает представление о связи между полом и риском заражения инфекционными заболеваниями, используя вирус Эбола в качестве африканского примера.

Методика: Всесторонний поиск литературы проводился с использованием баз данных PABMed, Ovid Medline и Global Health CABI, а также аннотаций CAB, включая "серую" литературу. Мы использовали описательный и гендерный анализ, чтобы по-новому взглянуть на предыдущие исследования вспышек лихорадки Эбола с 1976 по 2014 год. Нам удалось выделить из имеющихся данных отдельные случаи заражения и показатели летальности в зависимости от пола и известных случаев первоначального инфицирования.

Результаты: В общей сложности во всех предыдущих эпидемиях лихорадки Эбола с 1976 по 2012 год погибло примерно 1530 человек, при этом вспышка 2014 года унесла жизни 11310. Повышенное воздействие на женщин может быть связано с тем, что они больше времени проводили дома, поскольку в их обязанности входил уход за больными. При этом повышенная уязвимость мужчин перед вирусом может быть связана с их обязанностями по уходу за домашним скотом и временем, проведенным вне дома, поскольку большинство известных случаев первоначального инфицирования произошли в процессе охоты. Мы представляем концептуальную модель взаимодействия факторов риска для лихорадки Эбола в африканском контексте.

Вывод: В настоящее время нет данных, связанных с биологическими различиями между женским и мужским полом, которые бы ускоряли передачу вируса Эбола и увеличивали уязвимость; скорее всего, различия кроются в степени воздействия вируса на мужчин и женщин. Поэтому гендерный фактор является важным фактором риска, который следует учитывать при разработке программ здравоохранения. Создание потенциала для эффективного предупреждения рисков является достойным вкладом в национальную и международную систему здравоохранения, который позволит точнее реагировать на чрезвычайные ситуации в будущем.

Translated from English version into Russian by datran, through

La perspectiva de género en el virus del Ébola utilizando un marco de gestión de riesgos y de salud de la población: una revisión de alcance

Miriam N. Nkangu, Oluwasayo A. Olatunde, Sanni Yaya

Resumen

Antecedentes: En los tres decenios transcurridos desde que se registró el primer caso del virus del Ébola, la mayoría de los casos índice conocidos se han atribuido regularmente a la caza de "carne de monte", y las mujeres han registrado de forma constante tasas de mortalidad relativamente altas en la mayoría de los brotes catastróficos. Este artículo analiza los factores de riesgo relacionados con el Ébola, que interactúan constantemente con los valores culturales, y proporciona una idea del vínculo entre el género y el riesgo de contraer enfermedades infecciosas, utilizando el virus del Ébola como ejemplo en África.

Método: Se realizó una búsqueda exhaustiva de la literatura utilizando las bases de datos PubMed, Ovid Medline y Global Health CABI, así como CAB Abstracts, incluyendo la literatura gris. Se utilizó un análisis descriptivo y de género y sexo para revisar estudios previos sobre brotes de Ébola desde 1976 hasta 2014 y se desagregaron los casos y las tasas de mortalidad según el género y las fuentes de los casos índice conocidos basándose en los datos disponibles.

Resultados: En total, aproximadamente 1 530 personas murieron en todos los brotes previos de Ébola entre 1976 y 2012 en comparación con más de 11 310 defunciones desde el brote de 2014. La mayor exposición de las mujeres se puede atribuir al tiempo que pasan en casa y a su responsabilidad por cuidar a los enfermos, mientras que la mayor vulnerabilidad de los hombres al virus se puede atribuir a su responsabilidad por el cuidado del ganado y el tiempo que pasan lejos del hogar, ya que la mayoría de los casos índice conocidos se han infectado en el proceso de caza. Presentamos un modelo conceptual de un círculo de factores de riesgo interrelacionados para el Ébola en el contexto africano.

Conclusión: Actualmente no hay pruebas relacionadas con las diferencias biológicas entre el sexo femenino y masculino que aumenten la transmisión del virus del Ébola y la vulnerabilidad al mismo; más bien, hay diferencias en el nivel de exposición entre hombres y mujeres. Por lo tanto, el género es un importante factor de riesgo a considerar en el diseño de los programas de salud. Aumentar la capacidad para una comunicación eficaz del riesgo es una inversión en salud pública y mundial que vale la pena para futuras respuestas de emergencia.

Translated from English version into Spanish by mariagloria02, through

TRANSLATORS WITHOUT BORDERS
